# Pretreatment values of bilirubin and albumin are not prognostic predictors in patients with advanced pancreatic cancer

**DOI:** 10.1002/cam4.1848

**Published:** 2018-11-26

**Authors:** Lanyun Feng, Shihui Gu, Peng Wang, Hao Chen, Zhen Chen, Zhiqiang Meng, Luming Liu

**Affiliations:** ^1^ Department of Integrative Oncology Fudan University Shanghai Cancer Center Shanghai China; ^2^ Department of Oncology, Shanghai Medical College Fudan University Shanghai China; ^3^ Department of Hand Surgery, Huashan Hospital Fudan University Shanghai China; ^4^ Key Laboratory of Hand Reconstruction Ministry of Health Shanghai China; ^5^ Shanghai Key Laboratory of Peripheral Nerve and Microsurgery Shanghai China

**Keywords:** albumin, bilirubin, pancreatic cancer, prognosis

## Abstract

**Purpose:**

To identify the pretreatment values of bilirubin and albumin and other serum biomarkers in predicting the prognosis for advanced pancreatic cancer.

**Methods:**

A total of 201 consecutive patients pathologically diagnosed as advanced pancreatic cancer were retrospectively reviewed. Tumor location, TNM classification, the level of baseline total bilirubin (TBIT), direct bilirubin (DBIT), albumin (ALB), globulin (GLOB), total protein (TP), ALB to GLOB ratio (AGR), CA19‐9, CA242, and CA50 were collected. The values of CA19‐9, CA242, and CA50 were divided into two groups according to the upper limit value which were 1000 U/mL, 150 IU/mL, and 500 IU/mL, respectively. The values of TBIL, DBIL, IBIL, ALB, GLOB, TP, ALB, and GLOB were divided into low and high groups according to the median. To investigate if the median was an effective discriminator in dividing these markers, the patients were divided into a test set (n = 100) and a validation set (n = 101). Kaplan‐Meier (K‐M) survival analysis and Cox regression analysis were performed to explore the potential relationship between them and overall survival (OS).

**Results:**

A K‐M survival analysis revealed that the investigated markers in test set, including TBIL, DBIL, IBIL, ALB, GLOB, TP, ALB, and GLOB, were not associated with the OS. The findings from the validation set were consistent with those in the test set. Factors with *P* value smaller than 0.1 in the univariate analysis along with the tumor location, CA19‐9, CA242, CA50, were entered into the multivariate analysis. A Cox regression analysis suggested that the cancerization at head of pancreas (*P* = 0.01) and a high level of CA19‐9 (*P* = 0.02) were independent prognostic indicators for poor OS of pancreatic cancer.

**Conclusions:**

Baseline bilirubin and serum proteins were not associated with the prognosis of advanced pancreatic cancer. Tumor location and level of CA19‐9 may serve as significant indicators for poor prognosis in those patients.

## 1 INTRODUCTION

Pancreatic cancer is one of the most common causes of cancer deaths across the world with an overall 5‐years survival rate of 8%.^1^ Due to concealed manifestations, most patients are diagnosed at an advanced stage and have extremely limited chance of curative resection or surgical cure. It has been reported that patients with curative resection have longer median survival times (11.5 vs 3.6 months) and higher 5‐year survival rates (10.1% vs 3.7%) than patients with inoperable pancreatic cancer.^2^ However, the 5‐year survival rate has been reported to be no more than 25%, which is poor even if the potentially curative surgery was performed on those patients.^3^

It is well known that the overall survival of pancreatic cancer patients is linearly associated with primary tumor stage, lymph node involvement, vascular invasion, tumor differentiation, tumor size, and distance metastasis. However, these well‐known prognostic factors rely mainly on surgical exploration. In recent decades, researchers have shown interest in finding some minimally invasive ways of predicting outcome and design treatment plans.^4^, ^5^, ^6^

Bilirubin and albumin (ALB) have been proposed as endogenous antioxidants that play a role in multiple physiological and pathological processes as well as exerting anti‐carcinogenic effects.^7^, ^8^, ^9^ A 10‐year longitudinal study of 5460 men and 4843 women revealed that high serum bilirubin (within normal range) was related to low cancer mortality.^10^ A prospective population‐based study highlighted that a higher level of ALB was associated with lower risks of breast cancer and cancer mortality.^11^ In Liu et al’s^12^ study, baseline ALB and bilirubin were suggested as prognosis predictors of non‐metastatic breast cancer. However, to date, the prognostic value of pretreatment bilirubin and ALB in advanced pancreatic cancer has not been fully explored.

Therefore, this study is conducted with the aim to identify whether baseline bilirubin and ALB have protective properties against advanced pancreatic cancer and whether they have the potential to act as predictors for survival and prognosis. In this study, the overall survival (OS) of advanced pancreatic cancer patients was compared using different levels of bilirubin and ALB. In addition, other blood test indicators, such as baseline total protein (TP), globulin (GLOB), ALB to GLOB ratio (AGR) as well as tumor biomarkers, including carbohydrate antigen (CA)19‐9, CA 50, CA 242, were also enrolled and studied.

## 2 METHODS

### 2.1 Subjects

This retrospective study was approved by institutional ethics committee of Fudan University Shanghai Cancer Center. From January 2012 to January 2014, the consecutive in‐patients in the Department of Integrative Oncology, Fudan University Shanghai Cancer Center, who were pathologically diagnosed with advanced pancreatic cancer according to the 7th edition of the Union for International Cancer Control (UICC) staging systems, were included in this study. All patients received gemcitabine‐based palliative chemotherapy. The exclusion criteria included: (a) refusal to follow‐up; (b) absence of data; (c) no pathologic diagnosis; (d) no informed consent; (e) patients diagnosed with obstructive jaundice (serum TBIL >34.2 μmol/L and serum DBIL >50% of TBIL).

The patients’ characteristics were extracted from the medical records, including sex, age, location of pancreatic tumor, the tumor‐node‐metastasis (TNM) stage, survival outcomes and the blood test (total bilirubin (TBIL), direct bilirubin (DBIL), indirect bilirubin (IBIL), TP, ALB, GLOB, AGR), and tumor biomarkers (CA19‐9, CA 242, CA50).

In total, 217 patients who were pathologically diagnosed with pancreatic cancer satisfied the inclusion criteria. Three patients who were lost during follow‐up, and 13 patients who were diagnosed with obstructive jaundice (with serum TBIL >34.2 μmol/L and serum DBIL >50% of TBIL) were excluded. Ultimately, 201 patients were enrolled in this study for final analysis.

Baseline levels of TBIL, DBIL, IBIL, TP, ALB, GLOB, and AGR were divided into high and low groups according to the respective median values. To investigate whether the median was an effective discriminator while dividing these markers between groups, the patients were randomly divided into a test set (n = 100) and a validation set (n = 101). A univariate analysis focusing on the association between the baseline value of TBIL, DBIL, IBIL, TP, ALB, GLOB and AGR and OS was performed over both the test and validation sets to investigate whether the findings were consistent across the two sets.

Tumor biomarkers, including CA19‐9, CA 242, CA50, were performed by radioimmunoassay (RIA). Since a large portion of the enrolled patients had baseline value of CA19‐9, CA 242, and CA50 over the upper limit which could be measured using laboratory methods (if the value was over the upper limit that could be measured by lab methods, the laboratory test report would show as “> value *A*” rather than a specific number), these baseline values were divided into two groups according to the upper limit value, which were 1000 U/mL, 150 IU/mL, and 500 IU/mL, respectively. All the relevant blood tests were performed in the university's department of clinical laboratory in accordance with institutional guidelines and regulations.

### 2.2 Follow‐up

All the patients with advanced pancreatic cancer in our hospital were followed up by our Follow‐Up Department. A telephone follow‐up call was made every 3 months and the follow‐up ended with the patient's death. The OS was defined as the period from an initial diagnosis to confirmed death or final follow‐up.

### 2.3 Statistical analysis

All quantitative data were presented as numbers (percentages) or medians (ranges) as specified. Prism GraphPad (version 6, La Jolla, CA) and SPSS software (version 19.0, Armonk, NY) were used to analyze the data. Student's *t* test and chi‐squared test were used to identify if there was any difference in the baseline patient characteristics and clinical features between test set and validation set. Kaplan‐Meier (K‐M) survival analysis was applied to determine the differences in OS between groups. A Cox regression analysis was performed for multivariate analysis, using the forward stepwise method. *P* < 0.05 was considered to be statistically significant.

## 3 RESULTS

### 3.1 Patient characteristics and clinical features

Among the 201 patients included in this study, 128 (63.7%) were male. The median age of the patients was 61 (ranging from 27 to 83). According to the UICC staging system, 77 (38.3%) were diagnosed with stage III pancreatic cancer and 124 (61.7%) were diagnosed with stage IV pancreatic cancer. Seventy‐five (37.3%) of the tumors occurred in the head of the pancreas, and 126 (62.7%) in the body and tail. All the median values of TBIL, DBIL, IBIL, TP, ALB, GLOB, and AGR were within the normal range. The maximum of TP, ALB, GLOB, and AGR was within the normal range or only slightly exceeded the upper limit of the normal range. One hundred and thirty‐three (56.2%) of the patients had CA 19‐9 ≥1000 IU/mL, 109 (54.2%) of the patients had CA 242 ≥150 IU/mL, 140 (69.7%) of the patients had CA 50 ≥500 IU/mL. The enrolled patients were randomly divided into a test set (100 patients) and a validation set (101 patients). As shown in Table 1, no statistically significant differences were found in the baseline patient characteristics and clinical features between test set and validation set.

**Table 1:** Basic characteristics of 201 pancreatic cancer patients

Characteristics	N = 201	Test set N = 100	Validation set N = 101	*P* value
Gender, n (%)				
Female	73 (36.3%)	37 (37%)	36 (35.6%)	0.88^a^
Male	128 (63.7%)	63 (63%)	65 (64.4%)	
Age (y)				
Median (range)	61 (27‐83)	61 (27‐82)	62 (28‐83)	0.72^b^
Tumor location, n (%)				
Body/tail	126 (62.7%)	63 (63%)	63 (62.4%)	1.00^a^
Head	75 (37.3%)	37 (37%)	38 (37.6%)	
TNM classification, n (%)				
Stage III	77 (38.3%)	41 (41%)	36 (35.6%)	0.47^a^
Stage IV	124 (61.7%)	59 (59%)	65 (64.4%)	
TBIL (μmol /L)				
Median (range)	10.3 (3.4‐30.1)	10.6 (4‐28.3)	10 (3.4‐30.1)	0.56^b^
DBIL (μmol /L)				
Median (range)	3.4 (1.1‐18.3)	3.5 (1.2‐17.1)	3.4 (1.1‐18.3)	0.94^b^
IBIL (μmol /L)				
Median (range)	6.8 (1.5‐21.2)	7.2 (1.6‐19.1)	6.5 (1.5‐21.2)	0.38^b^
TP (g/L)				
Median (range)	68 (4.1‐90)	69.4 (4.1‐90)	66.9 (4.2‐82.4)	0.17^b^
ALB (g/L)				
Median (range)	41.4 (31.6‐51.3)	41.8 (33.6‐50)	41.4 (31.6‐51.3)	0.82^b^
GLOB (g/L)				
Median (range)	27 (17.4‐55.6)	27.7 (17.4‐55.6)	26.5 (19.1‐40.9)	0.09^b^
AGR				
Median (range)	1.5 (0.6‐2.3)	1.5 (0.6‐2.2)	1.5 (0.8‐2.3)	0.19^b^
CA19‐9(U/mL), n (%)				
<1000 U/mL	113 (56.2%)	55 (55%)	57 (56.4%)	0.89^a^
≥1000 U/mL	88 (47.8%)	45 (45%)	44 (43.6%)	
CA242(U/mL), n (%)				
<150 U/mL	109 (54.2%)	54 (54%)	54 (53.5%)	1.00^a^
≥150 U/mL	92 (45.8%)	46 (46%)	47 (46.5%)	
CA50(U/mL), n (%)				
<500 U/mL	140 (69.7%)	67 (67%)	73 (72.3%)	0.45^a^
≥500 U/mL	61 (30.3%)	33 (33%)	28 (27.7%)	

AGR, albumin‐globulin ratio; ALB, albumin; DBIL, direct bilirubin; GLOB, globulin; TBIL, total bilirubin; TP, total protein.

a Chi‐squared test.

b Student's *t* test.

### 3.2 Univariate analysis of prognostic factors in advanced pancreatic cancer in the test set

The test dataset was used for the identification of the prognostic markers. In the test set, the baseline levels of age, TBIL, DBIL, IBIL, TP, ALB, GLOB, and AGR were divided into high and low groups according to the median values (Table 1). The K‐M survival analysis was applied on these markers to investigate if they correlated with OS. The results identified that these markers, including TBIL (log rank = 0.45, *P* > 0.05), DBIL (log rank = 3.51, *P* > 0.05), IBIL (log rank = 0.88, *P* > 0.05), TP (log rank = 0.28, *P* > 0.05), ALB (log rank = 0.17, *P* > 0.05), GLOB (log rank = 0.22, *P* > 0.05), AGR (log rank = 0.74, *P* > 0.05), were not significantly associated with OS.

### 3.3 Univariate analysis for the identification of prognostic factors in advanced pancreatic cancer in the validation set

**Figure 1:**
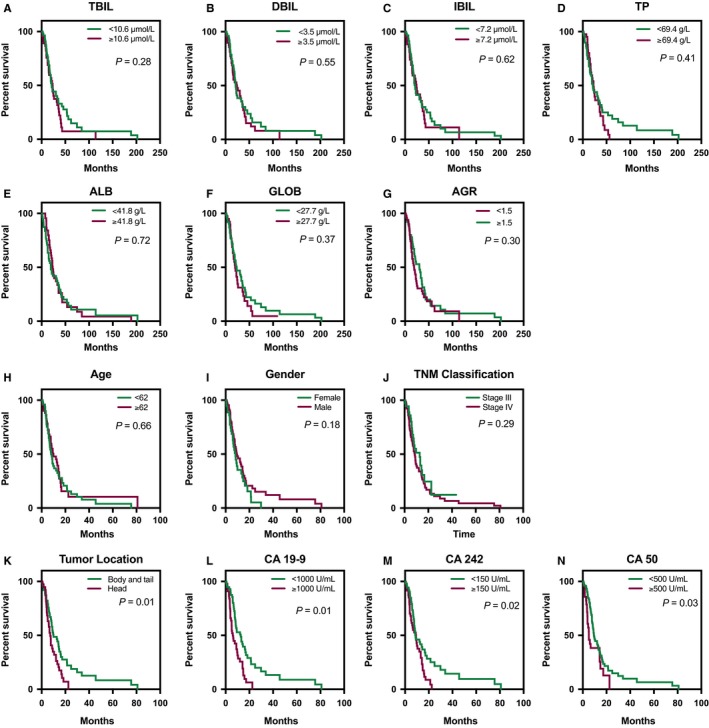
Kaplan‐Meier survival curves for overall survival (OS) in patients with pancreatic cancer after diagnoses in the validation set. A‐G, The OS in patients with high or low baseline value of total bilirubin (TBIL), direct bilirubin (DBIL), indirect bilirubin (IBIL), total protein (TP), albumin (ALB), globulin (GLOB), and albumin‐globulin ratio (AGR) was not different between the two groups (all *P* > 0.05); H‐J, The OS in patients with different age, gender, and TNM stage was not different between the two groups (all *P* > 0.05); K, The OS in patients with pancreatic head cancer was significantly longer than that in patients with tumor in pancreatic body and tail (*P* = 0.01); L, The OS in patients with baseline CA 19‐9 <1000 U/mL was significantly longer than that in patients with baseline CA 19‐9 ≥1000 U/mL (*P* = 0.01); M,The OS in patients with baseline CA 242 <150 U/mL was significantly longer than that in patients with baseline CA 242 ≥150 U/mL (*P* = 0.02); N,The OS in patients with baseline CA 50 <500 U/mL was significantly longer than that in patients with baseline CA 50 ≥500 U/mL (*P* = 0.03)

The markers in the validation set, including age, TBIL, IBIL, TP, ALB, GLOB, and AGR, were divided into high and low groups in accordance with the cutoff points identified in the test set. Similar to the findings from the test set, no markers were found to have the potential to predict survival time of patients with advanced pancreatic cancer (log rank = 1.66 and *P* > 0.05 for TBIL, log rank = 0.35 and *P* > 0.05 for DBIL, log rank = 0.24 and *P* > 0.05 for IBIL, log rank = 0.69 and *P* > 0.05 for TP, log rank = 0.13 and *P* > 0.05 for ALB, log rank = 0.81 and *P* > 0.05 for GLOB, log rank = 1.09 and *P* > 0.05 for AGR) (Table 2 and Figure 1).

**Table 2:** Univariate analyses for the association between clinical characteristics and survival in pancreatic cancer patients in the validation set

	N (%)	HR	95% CI	*P* value
Univariate analysis				
Gender, n (%)				
Female	36 (35.6)	1	Reference	
Male	65 (64.4)	1.35	0.87‐2.23	0.18
Age				
<62	50 (49.5)	1	Reference	
≥62	51 (50.5)	0.91	0.59‐1.4	0.66
Tumor location, n (%)				
Body/tail	63 (62.4)	1	Reference	
Head	38 (37.6)	1.73	1.17‐3.03	0.01
TNM classification, n (%)				
Stage III	36 (35.6)	1	reference	
Stage IV	65 (64.4)	1.29	0.81‐2.04	0.29
TBIL				
<10.6 μmol/L	55 (54.5)	1	Reference	
≥10.6 μmol/L	46 (45.5)	1.27	0.81‐1.98	0.28
DBIL				
<3.5 μmol/L	52 (51.5)	1	Reference	
≥3.5 μmol/L	49 (48.5)	1.14	0.74‐1.77	0.55
IBIL				
<7.2 μmol/L	58 (57.4)	1	Reference	
≥7.2 μmol/L	43 (42.6)	1.12	0.71‐1.75	0.62
TP				
<69.4 g/L	59 (58.4)	1	Reference	
≥69.4 g/L	42 (41.6)	1.20	0.76‐1.89	0.41
ALB				
<41.8 g/L	55 (54.5)	1	Reference	
≥41.8 g/L	46 (45.5)	0.92	0.60‐1.43	0.72
GLOB				
<27.7 g/L	61 (60.4)	1	Reference	
≥27.7 g/L	40 (39.6)	1.22	0.77‐1.94	0.37
AGR				
<1.5	50 (49.5)	1	Reference	
≥1.5	51 (50.5)	0.80	0.51‐1.23	0.30
CA19‐9				
<1000 U/mL	57 (56.4)	1	Reference	
≥1000 U/mL	44 (43.6)	2.07	1.48‐3.82	0.01
CA242				
<150 U/mL	54 (53.5)	1	Reference	
≥150 U/mL	47 (46.5)	1.64	1.12‐2.75	0.02
CA50				
<500 U/mL	73 (72.3)	1	Reference	
≥500 U/mL	28 (27.7)	1.67	1.06‐3.27	0.03
Multivariate analysis				
Tumor location, n (%)				
Body/tail	63 (62.4)	1	Reference	
Head	38 (37.6)	1.83	1.16‐2.90	0.01
CA19‐9				
<1000 U/mL	57 (56.4)	1	Reference	
≥1000 U/mL	44 (43.6)	2.87	1.22‐6.71	0.02
CA242				
<150 U/mL	54 (53.5)	1	Reference	
≥150 U/mL	47 (46.5)	0.89	0.41‐1.92	0.76
CA50				
<500 U/mL	73 (72.3)	1	Reference	
≥500 U/mL	28 (27.7)	0.79	0.41‐1.52	0.48

AGR, albumin‐globulin ratio; ALB, albumin; CI, confidence interval; DBIL, direct bilirubin; GLOB, globulin; HR, hazard ratio; IBIL, indirect bilirubin; TBIL, total bilirubin; TP, total protein.

### 3.4 Multivariate regression analysis for the identification of prognostic factors in advanced pancreatic cancer in the validation set

Univariate analyses were then performed on the validation set. The statistical results are shown in Table 2. Factors with *P *values smaller than 0.1 in the univariate analysis were entered into the multivariate analysis. The analysis indicated that cancerization at the head of pancreas and a baseline level of CA19‐9 ≥1000 IU/mL was independent predictors of poor OS in advanced pancreatic cancer. However, CA 242, CA50 were not independent factors in the prognosis of pancreatic cancer when adjusted for other parameters with multivariate analysis.

## 4 DISCUSSION

In this study, 13 of the 214 patients had baseline bilirubin levels greater than the upper limit of normal range and were diagnosed as obstructive jaundice. In these patients, increased deposition of bilirubin in the liver may have damaged the liver cells, causing liver dysfunction or, even, liver failure. In addition, bilirubin can freely pass into the brain interstitium and induce neurotoxicity. These pathological processes may affect survival in patients with advanced pancreatic cancer, leading to a poor prognosis. Nakata et al^13^ have demonstrated that the presence of obstructive jaundice at diagnosis in patients with pancreatic head cancer may predict an unfavorable survival compared to such patients without obstructive jaundice. To avoid the interferences caused by high levels of bilirubin, those 13 patients with bilirubin levels exceeding normal range were excluded in this study. The results of this study suggest that there is no relationship either between TBIL, DBIL or IBIL and OS. Although bilirubin was proposed in Kühn et al’s^11^ study as an endogenous antioxidant as having anti‐carcinogenic effects, serum bilirubin level was not associated with end points of breast cancer, prostate cancer, lung cancer, and colorectal cancer. More studies are required to identify the role of bilirubin, an endogenous antioxidant, in pancreatic cancer.

ALB is the main protein of human plasma. The main function of ALB is to maintain the oncotic pressure of blood. ALB has also been identified as an important endogenous antioxidant. In addition, ALB is an important indicator of nutritional status. Low level of ALB has been found to be a solid risk factor for poor prognosis of pancreatic cancer. Ruiz‐Tovar et al^14^ analyzed 59 patients and reported that preoperative level of ALB less than 28 g/L predicted a worse chance of survival. Siddiqui et al^15^ also reported that a lower level (32 g/L) of ALB is associated with a shorter period of survival. In this study, no relationship was found between OS and baseline ALB in pancreatic cancer patients in either the test set or the validation set. This negative result can be interpreted by the relatively high level of ALB in those patients. In our study, the median baseline ALB level of all the 201 patients enrolled was 41.4 g/L, which was within the normal range (40‐55 g/L) and was also higher than the level reported by Ruiz‐Tovar and Siddiqui et al^14^, ^15^ GLOB is another important component of total serum protein, which plays an important role in immunity and inflammation systems. Some immune globulins, such as gamma globulins, are immunoglobulins, also known as antibodies. However, its role in cancer suppression has not yet been well investigated. In Liu et al’s^16^ study, no relationship was found between baseline GLOB level and OS in patients with gastric cancer. In this study, K‐M survival analysis in both its test and validation sets showed little association between baseline GLOB and OS. In addition to ALB and GLOB, the level of AGR was also analyzed. It has been reported that a low level of AGR has the potential to be a predictor for poor prognosis for breast cancer,^17^ gastric cancer,^16^, ^18^ colorectal cancer,^19^ and lung cancer.^20^, ^21^ However, in this study, the median of AGR was 1.5 (ranging from 0.6 to 2.3). No obvious decrease was detected in AGR and no association was found between AGR and the prognosis.

Tumor biomarkers can also potentially act as a prognostic predictor of pancreatic cancer. Mucins are a family of glycosylated proteins with high molecular weight. In pancreas, mucins are secreted by pancreatic duct cells.^22^ It has been reported that mucin gene is abnormally expressed in pancreatic cancer and may have opportunities to play a role in the diagnosis and therapy.^23^ CA 19‐9, CA 242, and CA 50 are mucin markers and are thought to be aberrantly expressed when patients are suffering from pancreatic cancer. In Lei et al’s^24^ study, the expression level of CA 19‐9, CA242, CA 50 was found to increase in patients with pancreatic cancer and similarly, the sensitivity of CA19‐9 and specificity of CA242 in detecting the tumor malignancy. Chen et al^6^ reported that higher CA242 and CA199 are associated with a lager tumor size, higher TNM stage as well as poor tumor differentiation. To date, there have been limited studies exploring the potential of these in predicting the prognosis in pancreatic cancer. In our study, the results from K‐M survival analysis suggested that lower levels of CA 19‐9, CA 242, and CA 50 are associated with a longer period of survival. However, our multivariate analysis showed that only CA 19‐9 is the independent prognostic factor for pancreatic cancer. These results echo the results from Gu et al’s study.^25^ In their study, higher levels of CA19‐9, CA125, CEA, and CA242 were found to be related to pancreatic cancer, but only CA19‐9 was the independent factor for prognosis prediction after the Cox regression analysis.^25^

In pancreatic cancer, whether the anatomic location of tumors has a significant prognostic impact, remains in conflict. Pancreatic cancers of the body and tail were found to have lower rates of survival.^26^, ^27^ However, in Liu et al and Wu et al’s study, no statistically significant relationship was found between tumor location and OS.^4^, ^28^ In this study, with the univariate analysis, cancerization at head of pancreas was statistically significantly associated with poor prognosis. Following multivariate analysis, cancerization at the head of pancreas was also found to be an independent prognostic factor for poor OS. It is known that a tumor located in the head of pancreas often causes bile obstruction. Bile obstruction will increase the level of bilirubin, which may damage the liver function. This might be the reason that pancreatic tumors located in the head were associated with poor survival in this study. More studies are required in this area.

This study has some limitations. First of all, since this is a retrospective cohort study, only Level III evidence was obtained. Second, all of the in‐patients with advanced pancreatic cancer in our hospital were treatable with minimally invasive treatment combined with systemic therapy. This selection bias might be the reason why patients with advanced pancreatic cancer had relatively normal levels of nutritional status. Third, this study was a single center study and the sample size was relatively small and the enrolled subjects were all from Chinese Han population. A multicenter prospective study with a larger sample size and longer follow‐up is required to confirm our findings.

## CONFLICT OF INTEREST

The authors declare no conflict of interests in this study.
